# Comparative efficacy of different exercise modalities on metabolic profiles and liver functions in non-alcoholic fatty liver disease: a network meta-analysis

**DOI:** 10.3389/fphys.2024.1428723

**Published:** 2024-09-11

**Authors:** Mingming Huang, Jiafa Yang, Yihao Wang, Jian Wu

**Affiliations:** ^1^ School of Exercise Science and Health, Capital University of Physical Education and Sports, Beijing, China; ^2^ School of Arts and Sports, Dong-A University, Busan, Republic of Korea

**Keywords:** nonalcoholic fatty liver disease, exercise, network meta-analysis, aerobic, physical activity

## Abstract

**Objective:**

Research evidence suggests that exercise is a potent therapeutic strategy for non-alcoholic fatty liver disease (NAFLD). Many investigations have delved into the curative potential of diverse exercise regimens on NAFLD. This investigation synthesizes findings from randomized controlled trials via a network meta-analysis to evaluate the efficacy of exercise-based interventions on NAFLD.

**Methods:**

We conducted a search across five electronic databases (Web of Science, EMBASE, PubMed, SCOPUS, and CNKI)to identify randomized controlled trials (RCTs) comparing the effects of different exercise modalities on metabolic profiles and liver functions in patients with NAFLD. The literature search was comprehensive up to 15, December 2023. The selected studies were subjected to a rigorous quality appraisal and risk of bias analysis in accordance with the Cochrane Handbook’s guidelines, version 5.1.0. We employed Stata/MP 17 for the network meta-analysis, presenting effect sizes as standardized mean differences (SMD).

**Results:**

This study aggregated results from 28 studies, involving a total of 1,606 participants. The network meta-analysis revealed that aerobic exercise was the most effective intervention for improving BMI in patients with NAFLD, demonstrating a significant decrease in BMI (−0.72, 95%CI: −0.98 to −0.46; *p* < 0.05; Surface Under the Cumulative Ranking (SUCRA) = 79.8%). HIIT was the top intervention for enhancing HDL-C (0.12, 95% CI: 0.04 to 0.20; *p* < 0.05; SUCRA = 76.1%). Resistance exercise was the most effective for reducing LDL-C (−0.20, 95% CI: −0.33 to −0.06; *p* < 0.05; SUCRA = 69.7%). Mind-body exercise showed superior effectiveness in improving TC (−0.67, 95% CI: −1.10 to −0.24; *p* < 0.05; SUCRA = 89.7%), TG = −0.67, 95% CI: −1.10 to −0.24; *p* < 0.05; SUCRA = 99.6%), AST (−8.07, 95% CI: −12.88 to −3.25; *p* < 0.05; SUCRA = 76.1%), ALT (−12.56, 95% CI: −17.54 to −7.58; *p* < 0.05; SUCRA = 99.5%), and GGT (−13.77, 95% CI: −22.00 to −5.54; *p* < 0.05; SUCRA = 81.8%).

**Conclusion:**

This network meta-analysis demonstrates that exercise interventions positively affect various metabolic profiles and liver functions in NAFLD patients. Mind-body exercises are particularly effective, surpassing other exercise forms in improving metabolic profiles and liver functions.

**Systematic Review Registration:**

https://www.crd.york.ac.uk/PROSPERO/, identifier registration number CRD42024526332.

## 1 Introduction

Non-alcoholic fatty liver disease (NAFLD) encompasses a spectrum of hepatic dysfunctions primarily driven by metabolic dysregulation (Powell et al., 2021). Intrinsically linked to obesity, metabolic syndromes, type 2 diabetes, and insulin resistance, NAFLD’s prevalence is notably increasing in parallel with obesity rates, currently estimated at 25% among adults (Younossi et al., 2018). Notably, a study projected an increase in NAFLD incidence by 13.5%–29.5% by 2030 across diverse national contexts including the United States, the United Kingdom, China, and Italy (Estes et al., 2018).

Sedentary lifestyles and poor dietary habits augment the potential risk of NAFLD, prompting researchers to explore interventions targeting modifiable factors. Lifestyle modifications have emerged as efficacious strategies in early NAFLD management (Ding et al., 2017), and adopting a Mediterranean dietary pattern shown to significantly reduce hepatic fat in overweight NAFLD patients (Trovato et al., 2015). Furthermore, prospective research indicates associations between sedentary behavior changes and alterations in waist circumference and cardiovascular metabolic risk scores (Healy et al., 2008), thus underscoring the importance of considering sedentary status alteration and increased physical activity as avenues of investigation. Exercise has been found to mitigate insulin resistance, type 2 diabetes, and obesity (Snowling and Hopkins, 2006; Romero-Gómez et al., 2017), exhibiting favorable effects on transaminase levels and dyslipidemia (Li and Huang, 2021bib_li_and_huang_2021). Even without weight loss, consistent exercise regimens have been shown to reduce hepatic fat by 20%–30% (Hashida et al., 2017). However, despite numerous clinical interventions and randomized controlled trials targeting NAFLD, the differential impacts of various exercise modalities (aerobic, resistance, high-intensity interval training (HIIT), and mind-body exercise) remain inconclusive.

In recent years, numerous studies have focused on the impact of various forms of exercise on NAFLD (Nam et al., 2023; Hejazi and Hackett, 2023; Chun, 2023). Research has highlighted the effects of aerobic exercise and resistance training in improving liver health and overall metabolic function in NAFLD patients. Nam et al. (2023), in a systematic review of 11 studies, explored the effects of different exercise modalities on intrahepatic lipid (IHL), alanine aminotransferase (ALT), body mass index (BMI), and insulin resistance (IR) in patients with NAFLD. Their findings indicate that aerobic exercise can significantly improve IHL and ALT levels. However, a critical review of these studies reveals a predominant focus on aerobic and resistance exercises, with a noticeable lack of comprehensive examination of other exercise modalities. Furthermore, these studies often combined the effects of different exercise interventions without directly comparing their efficacy. This indicates a gap in the literature, underscoring the need for more detailed comparative studies on various exercise forms in the management of NAFLD.

Liver function and lipid biomarkers serve as common indicators for evaluating the severity of NAFLD and associated metabolic abnormalities (Wong et al., 2018). Within lipid metabolism indicators, total cholesterol (TC), triacylglycerol (TG), and low-density lipoprotein cholesterol (LDL-C) are commonly utilized to assess the severity of NAFLD and its close association with metabolic abnormalities (Wong et al., 2018; Vilar-Gomez and Chalasani, 2018). Low levels of high-density lipoprotein cholesterol (HDL-C) are frequently observed in lipid metabolism disorders (Fan et al., 2011) and are closely linked to the progression of NAFLD (Feng et al., 2020; Mato et al., 2019). Aspartate aminotransferase (AST) and ALT serve as sensitive indicators of hepatic cell damage (Senior, 2012), while sustained elevation of gamma-glutamyl transferase (GGT) may indicate progression to chronic hepatitis and is an important marker for identifying NAFLD (Kunutsor, 2016; Ha et al., 2022). BMI is considered the most commonly used measure for defining obesity and overweight populations across various genders and age groups (Daniels, 2009). Due to the close relationship between obesity and NAFLD, there are studies suggesting that BMI might be an important method for identifying NAFLD (Camhi et al., 2011). In summary, in our study, we selected eight outcome indicators closely related to NAFLD and aimed to provide relatively effective exercise modalities for adult NAFLD patients through network meta-analysis.

## 2 Methods

This study adheres to the Preferred Reporting Items for Systematic Reviews and Meta-Analyses (PRISMA) guidelines (Moher et al., 2010), with the research protocol prospectively registered in the PROSPERO database (https://www.crd.york.ac.uk/PROSPERO/, CRD42024526332).

### 2.1 Search strategy

The literature search was conducted using Web of Science, EMBASE, PubMed, SCOPUS, and China National Knowledge Infrastructure (CNKI) databases up to December 15, 2023, to identify randomized controlled trials. Keywords such as “exercise,” “obesity,” “lifestyle,” “physical activity,” “aerobic,” “liver,” and “NAFLD” were combined using the “OR” operator. Specific search strategies included.

• Web of Science: TS=(“exercise” OR “physical activity” OR “aerobic” OR “lifestyle”) AND TS=(“obesity” OR “NAFLD” OR “liver”)
• EMBASE: “exercise”/exp OR “physical activity”/exp OR “aerobic exercise”/exp OR “lifestyle”/exp AND “obesity”/exp OR “nonalcoholic fatty liver disease”/exp OR “liver disease”/exp
• PubMed: (“exercise” [MeSH Terms] OR “physical activity” [MeSH Terms] OR “aerobic exercise” [MeSH Terms] OR “lifestyle” [MeSH Terms]) AND (“obesity” [MeSH Terms] OR “NAFLD” [MeSH Terms] OR “liver” [MeSH Terms])
• SCOPUS: TITLE-ABS-KEY (“exercise” OR “physical activity” OR “aerobic” OR “lifestyle”) AND TITLE-ABS-KEY (“obesity” OR “NAFLD” OR “liver”)
• CNKI: TS=(“exercise” OR “physical activity” OR “aerobic” OR “lifestyle”) AND TS=(“obesity” OR “NAFLD” OR “liver”)

Detailed search terms are documented in Supplementary Material S1.

### 2.2 Inclusion and exclusion criteria

Inclusion criteria: (1) Randomized controlled trials (RCTs) that investigate the use of exercise therapy in patients with NAFLD; (2) Subjects diagnosed with NAFLD via histopathological or imaging examinations; (3) No significant differences in baseline values of outcome indicators before intervention in patients; (4) Outcome indicators include BMI, TG, TC, LDL-C, HDL-C, ALT, AST, and GGT; (5) The type of intervention must be one of the following: aerobic exercise, resistance exercise, HIIT, or mind-body exercise; (6) The exercise intervention period must last at least 4 weeks.

Exclusion criteria: (1) Specific data on outcome indicators (such as AST, ALT, TC, TG, BMI, etc.) are unavailable. (2) Excluded studies include animal experiments, abstracts, case reports, reviews, systematic evaluations, and duplicate publications. (3) Studies where the type, duration, and frequency of exercise are unclear. (4) Studies focusing only on lifestyle changes without specific exercise regimens.

### 2.3 Data extraction and quality assessment

The data extraction process included relevant information from the literature, such as the title, authors, publication year, journal, number of patients, age, gender, intervention measures, and outcome indicators. The intervention measures encompassed four types of exercise modalities: aerobic, resistance, HIIT, and mind-body exercise. The aerobic exercise included brisk walking, cycling, jogging, and swimming, while mind-body exercise involved yoga, tai chi, baduanjin, and Wushu qigong. Definitions for each intervention modality are provided in Supplementary Material S2. Outcome indicators included BMI, lipid metabolism indicators, and liver function markers. Lipid metabolism indicators comprised TC, HDL-C, TG, and LDL-C. Liver function markers included GGT, AST, and ALT. Extracted data were presented as mean ± standard deviation (SD) before and after the intervention. Data presented as median and quartiles were converted to mean ± SD.

We conducted a comprehensive assessment of potential biases in the included studies using the risk of bias assessment tool recommended in the Cochrane Handbook 5.1.0 (Higgins et al., 2011). Data extraction and risk assessment were independently performed by two individuals. To maintain objectivity, any discrepancies during the screening process were resolved through discussion with a third researcher.

### 2.4 Statistical analysis

A frequency-based network meta-analysis of outcome indicators was conducted using Stata 17.0 software. For continuous variables, the mean difference (MD) was estimated via network meta-analysis. Given the consistency of outcome indicator units, MD and SD were used to calculate indicators. All meta-analysis results are thoroughly detailed in the results section. Network plots for various outcome indicators were constructed using the “network” command, where nodes represent different intervention methods. Node size correlates with the sample size of each treatment method, with larger nodes denoting greater sample sizes. The thickness of the lines between nodes indicates the number of studies, whereas thicker lines signify more studies. The SUCRA was used to determine the probability ranking of different exercise patterns as the optimal intervention method. A higher SUCRA value indicates a higher probability that a specific intervention will be the most effective (Rücker and Schwarzer, 2015).

Comparative funnel plots were used to assess publication bias. In cases where closed loops were present, inconsistency tests were conducted. When the inconsistency tests yielded *p* < 0.05, significant inconsistencies between direct and indirect comparison results were noted. Two levels of inconsistency assessment were conducted: global and local. Overall inconsistency was evaluated first, followed by node-splitting methods to assess local inconsistencies. The global I^2^ statistic was used to evaluate heterogeneity; values exceeding 75% indicated substantial heterogeneity. A random-effects network meta-analysis model was used in cases of high heterogeneity, while a fixed-effect model was employed for lower heterogeneity.

## 3 Results

### 3.1 Search results

**FIGURE 1:**
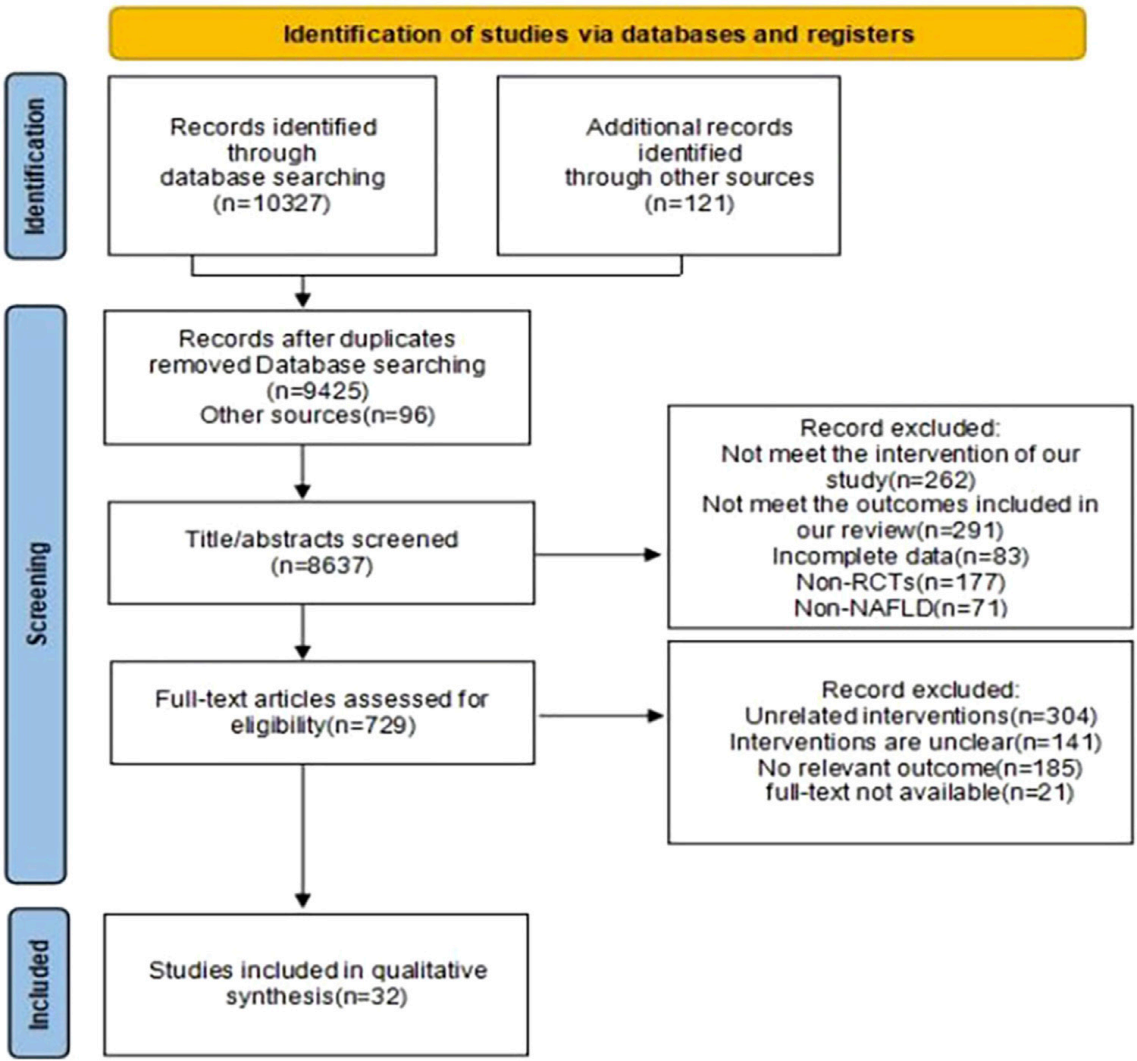
Flowchart of the screening process.

Our search identified 10,448 articles. Through processes including duplicate removal, abstract reviews, and full-text examinations, we excluded studies that failed to meet our inclusion criteria. Ultimately, 28 studies fulfilling the eligibility requirements were selected for analysis, The specific retrieval process is depicted in Figure 1. Of these, four studies compared two distinct exercise interventions (Cai, 2023; Fu et al., 2018; Yang, 2016; Winn et al., 2018). These studies were grouped into two separate groups, A and B, resulting in a total of 32 interventions, though the number of articles analyzed remained at 28. The included studies comprised 23 on aerobic exercise, six on resistance exercise, five on HIIT, and three on mind-body exercises. This collection included two studies comparing aerobic to resistance exercises, two comparing aerobic to HIIT, and one comparing aerobic to mind-body exercises. In total, 1,606 participants were involved in the included trials, with only two studies having a duration of less than 8 weeks. The basic characteristics of the included studies are presented in Table 1.

**TABLE 1:** Basic characteristics of the included literature.

Author (year)	Control group				Experiment group				Duration (week)	Outcomes
	Number	Age	Intervention type	Intensity	Number	Age	Intervention type	Intensity		
Wang et al. (2023)	21	19.33 ± 1.35	Aerobic	60% −70%HR max	22	19.50 ± 1.44	HIIT	90% −95%HRmax	8	①②③④⑤
Yang et al. (2015)	48	48.4 ± 4.8	No intervention	—	48	47.1 ± 3.9	Aerobic	Low to moderate intensity	24	①②③④⑤
Tan et al. (2015)	19	46–59	No intervention	—	18	46–59	Aerobic	FATmax	24	①②③④⑤
Liu et al. (2014)	22	12.87 ± 1.46	Medication	—	22	13.02 ± 1.73	Medication Aerobic	140-160bpm/min	12	①②③④⑤⑥⑦
Peng et al. (2022)	27	21.8 ± 0.8	No sports	—	27	21.3 ± 1.0	Dietary Aerobic	FATmax	12	①②③④⑤⑥⑦⑧
Liu et al. (2019)	30	61.5 ± 8.2	Medication	—	30	60.5 ± 8.5	Medication Aerobic	Moderate intensity	16	②③④⑤⑦
Wang (2006)	29	49.17 ± 8.71	No sports	—	42	51.93± 7.68	Aerobic	40%-60%HRmax	12	①②③④⑤
Babu et al. (2022)	22	56.7 ± 10.7	No intervention	—	20	59.9 ± 9.8	HIIT	85% VO_2_max	12	①②③④⑤⑥⑦⑧
Cuthbertson et al. (2016)	31	46–59	No intervention	—	38	46–58	Aerobic	30%-60%HRR	16	①②③④⑤⑥⑦⑧
Charatcharoenwitthaya et al. (2021)	19	37.4 ± 1.9	Aerobic	60%–70% HRmax	19	38.2 ± 2.2	Resistance	60% 1RM	12	①②③④⑤⑥⑦⑧
De Piano et al. (2012)	14	15–19	Aerobic	50%–70% VO_2_max	14	15–19	Resistance	Low to moderate intensity	48	①②③④⑤⑥⑦⑧
Abdelbasset et al. (2019)	16	55.2 ± 4.3	Medication	—	16	54.4 ± 5.8	HIIT	80%-85%VO_2_max	8	②④⑤⑦
Sullivan et al. (2012)	6	47.5 ± 3.1	No intervention	—	12	48.6 ± 2.2	Aerobic	Low to moderate intensity	16	①②③④⑤⑥⑦
Sánchez-Muñoz (2013)	8	25–60	Medication	—	8	25–60	Aerobic	60–85%HRmax	12	①②③④⑤⑥⑦
Pugh et al. (2013)	6	50 ± 3	Routine nursing	—	5	50 ± 3	Aerobic	Moderate intensity	16	①②③④⑤⑥⑦⑧
Pugh et al. (2014)	8	47.0 ± 2.2	Routine nursing	—	13	48.2 ± 2.1	Aerobic	Moderate intensity	16	①②③④⑤⑥⑦⑧
Rezende et al. (2016)	21	54.5 ± 8.9	No intervention	—	19	56.2 ± 7.8	Aerobic	Moderate intensity	24	①②③④⑤⑥⑦⑧
Shojaee-Moradie et al. (2016)	12	52.8 ± 3.0	Lifestyle advice	—	15	52.4 ± 2.2	Aerobic	40–60%HRmax	16	①②③④⑤⑥⑦⑧
Dong et al. (2016)	130	57.94 ± 5.71	No intervention	—	130	56.68 ±5.33	Aerobic	60%–80% target heart rate	12	①②③④⑤⑥⑦⑧
Zelber-Sagi et al. (2014)	31	46.6 ± 11.4	Home stretching	—	33	46.32 ± 10.32	Resistance	Moderate intensity	12	①②③④⑤⑥⑦⑧
Zhu et al. (2020)	48	45 ± 3	Medication	—	48	44 ± 4	Medication Mind-body	30%–50% VO_2_max	12	②③④⑤⑥⑦⑧
Zhu (2022)	30	47.6 ± 12.38	Aerobic	60%–70% HRmax	30	45.96 ± 12.82	Mind-body	30%–50% VO_2_max	12	②③⑥⑦
Pang and Jin (2023)	30	62.0± 13.46	Medication	—	30	61.25 ± 14.65	Medication Mind-body	30%–50% VO_2_max	12	②③⑥⑦⑧
Cai (2023) A	6	52.33 ± 9.69	Dietary control	—	6	53.17 ± 9.24	Aerobic	FATmax	12	①②③④⑤⑥⑦⑧
Cai (2023) B	6	52.33 ± 9.69	Dietary control	—	6	45.5 ± 7.34	Resistance	8-12RM	12	①②③④⑤⑥⑦⑧
Fu et al. (2018)	30	58.16 ± 9.80	Lifestyle advice	—	28	61.18 ± 7.53	Aerobic	60%-70%HRmax	16	①②③④⑤⑦
Fu et al. (2018)	30	58.16 ± 9.80	Lifestyle advice	—	27	55.90 ± 12.30	Resistance	60%–80%1RM	16	①②③④⑤⑦
Yang (2016)	31	58.06 ± 9.79	Lifestyle advice	—	29	61.28 ± 7.52	Aerobic	60%-70%HRmax	20	①②③④⑤⑦
Yang (2016)	31	58.06 ± 9.79	Lifestyle advice	—	28	55.80 ± 12.29	Resistance	50%–60%1RM	20	①②③④⑤⑦
Abdelbasset et al. (2020)	16	55.2 ± 4.3	Lifestyle advice	—	16	54.4 ± 5.8	HIIT	80%-85%VO_2_max	8	①②③④⑤⑦
Winn et al. (2018)	5	51 ± 13	No intervention	—	8	46 ± 9	Aerobic	55%VO_2_peak	4	①②③④⑤⑦
Winn et al. (2018)	8	46 ± 9	Aerobic	55%VO_2_peak	8	41 ± 14	HIIT	80%VO_2_peak	4	①②③④⑤⑦

A and B, intervention studies with different exercise modalities in the same trial; ①, body mass index (BMI); ②, total cholesterol (TC); ③, triacylglycerol (TG); ④, high-density lipoprotein cholesterol (HDL-C); ⑤, low-density lipoprotein cholesterol (LDL-C); ⑥, aspartate aminotransferase (AST); ⑦alanine aminotransferase (ALT); ⑧, gamma-glutamyl transferase (GGT); FATmax, maximal fat oxidation; VO2max, maximum rate of oxygen consumption; HRR, heart rate reserve; VO_2_peak, peak oxygen consumption; 1RM, one-repetition maximum; Lifestyle advice, these include adherence to doctor’s instructions, dietary health education, organizing NAFLD, health seminars, and conducting monthly follow-up phone calls.

### 3.2 Risk of bias

**FIGURE 2:**
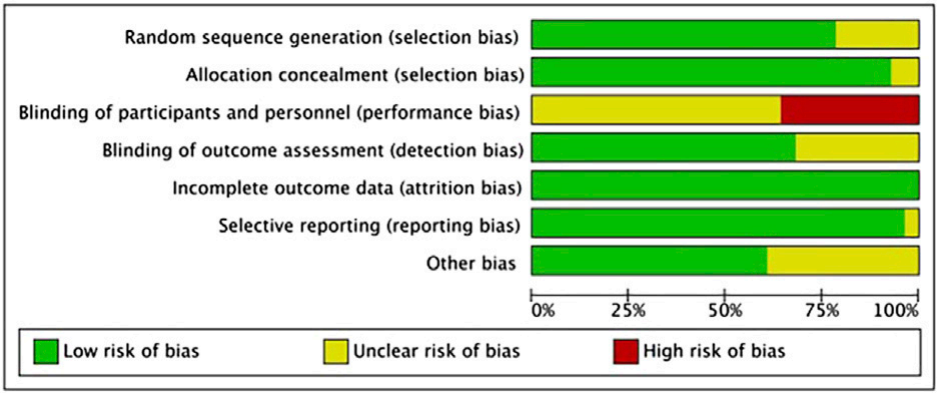
Schematic of Cochrane bias risk assessment.

This investigation incorporated 28 RCTs. Among these, 22 studies clearly described the methods used for generating random sequences, and two studies implemented appropriate allocation concealment. On participants and personnel blinding, considering the nature and type of interventions, blinding of participants and controllers was challenging; however, an overall assessment indicated a generally low risk of bias (refer to Figure 2). Risk of bias in individual studies in Supplementary Material S3.

### 3.3 Network meta-analysis

**FIGURE 3:**
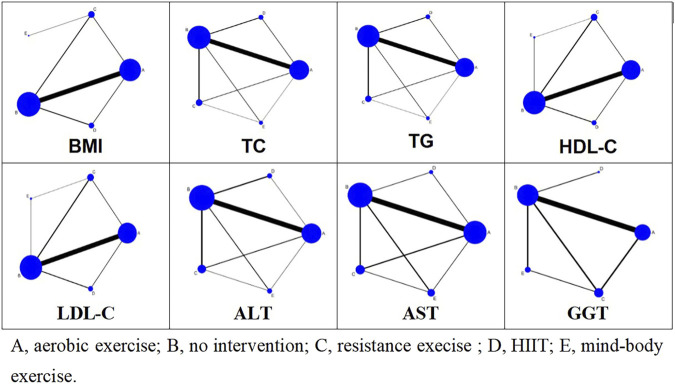
Network meta-analysis maps of four types of exercise interventions.

Figure 3 illustrates the network relationships among four types of exercise interventions and a control intervention. In the diagram, “A” represents aerobic exercise, “B” denotes no intervention, “C” signifies resistance exercise, “D” stands for HIIT, and “E” indicates mind-body exercise. Each intervention type is represented by a node, with lines connecting these nodes to depict direct comparisons made in studies. The absence of a line between any two nodes suggests no direct comparative research exists for these specific interventions within the reviewed literature. The size of each node reflects the number of studies associated with each intervention type, with larger nodes indicating a greater number of studies. Similarly, the thickness of the lines between two nodes correlates with the number of studies directly comparing these interventions.

#### 3.3.1 Effects of exercise interventions on BMI

**FIGURE 4:**
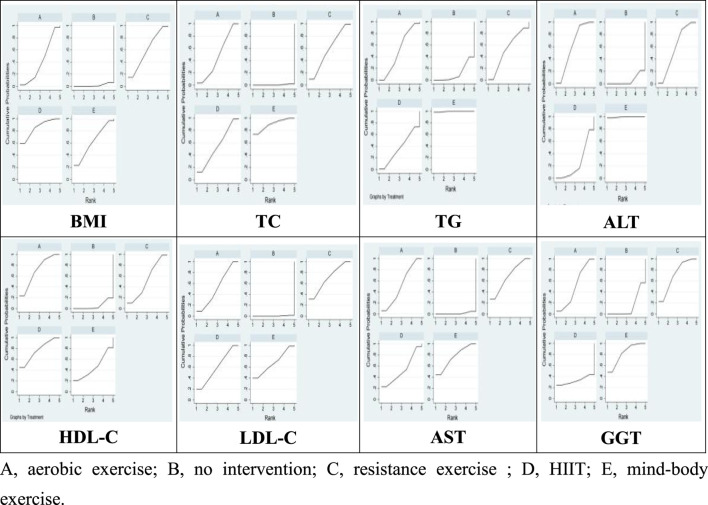
SUCRA ranking chart.

28 studies were included in this meta-analysis to investigate the effects of various interventions on the BMI of patients diagnosed with NAFLD. The findings from pairwise meta-analyses indicated that, compared to the control group, aerobic exercise (−0.72, 95% CI: −0.98 to −0.46; *p* < 0.05), HIIT (−0.65, 95% CI: −1.09 to −0.20; *p* < 0.05) were associated with significant improvements in BMI among NAFLD patients’ post-intervention. However, the effect of mind-body exercise (−0.26, 95% CI: −1.15 to 0.63; *p* > 0.05), and resistance exercise (−0.51, 95% CI: −1.37 to 0.35; *p* > 0.05) on BMI was not statistically significant. Furthermore, the SUCRA analysis, based on the consistency model, revealed that aerobic exercise SUCRA scores of 79.8, and HIIT SUCRA scores of 69.0 exhibited superior efficacy in improving BMI among NAFLD patients, while mind-body exercise SUCRA scores of 27.2, and resistance exercise SUCRA scores of 63.8 ranked the low in terms of effectiveness Table 2 and Figure 4). These results suggest that aerobic exercise may be more beneficial for managing BMI in NAFLD patients compared to mind-body exercise.

**TABLE 2:** Basic characteristics of the included literature.

Treatment type	Comparison to no treatment		Best	Worst	SUCRA(%)
	SMD (95% CI)	*P*-Value			
BMI					
Aerobic exercise	−0.7 (-0.98, -0.46)	<0.05	44.9	0.0	79.8
Resistance exercise	−0.51 (-1.37, 0.35)	>0.05	27.1	0.1	63.8
HIIT	−0.6 (-1.09, -0.20)	<0.05	27.5	0.1	69.0
Mind-body exercise	−0.26 (-1.15, 0.63)	>0.05	0.5	28.6	27.2
TC					
Aerobic exercise	−0.3 (-0.55, −0.16)	<0.05	3.4	0.0	47.5
Resistance exercise	−0.41 (−0.74, −0.08)	<0.05	10.0	0.8	57.5
HIIT	−0.39 (−0.74, −0.04)	<0.05	12.6	1.4	54.7
Mind-body Exercise	−0.67 (−1.10, −0.24)	<0.05	74.0	0.1	89.7
TG					
Aerobic exercise	−0.33 (−0.72, 0.05)	>0.05	0.1	2.7	50.2
Resistance exercise	−0.41 (−0.74, −0.08)	<0.05	0.7	10.5	52.2
HIIT	−0.39 (−0.74, −0.04)	<0.05	0.8	26.6	36.5
Mind-body exercise	−0.67 (−1.10, −0.24)	<0.05	98.4	0.0	99.6
HDL-C					
Aerobic exercise	0.67 (0.24, 1.10)	<0.05	23.9	0.0	70.3
Resistance exercise	0.08 (0.00, 0.16)	<0.05	10.4	1.1	52.7
HIIT	0.12 (0.04, 0.20)	<0.05	44.8	0.3	76.1
Mind-body exercise	0.07 (−0.08, 0.22)	>0.05	20.9	18.4	45.6
LDL-C					
Aerobic exercise	−0.16 (−0.24, 0.09)	>0.05	8.9	0.0	52.6
Resistance exercise	−0.20 (−0.33, −0.06)	<0.05	31.2	0.1	69.3
HIIT	0.04 (−0.07, 0.15)	>0.05	19.9	0.1	60.0
Mind-body exercise	−0.01 (−0.15, 0.13)	<0.05	40.0	1.4	67.7
ALT					
Aerobic exercise	−5.98 (−9.05, −2.91)	<0.05	0.8	0.0	62.1
Resistance exercise	−5.66 (−9.93, −1.39)	<0.05	0.8	0.3	58.0
HIIT	−2.03 (−7.08, 3.01)	>0.05	0.1	21.3	25.0
Mind-body exercise	−12.56 (-17.54,-7.58)	<0.05	98.3	0.0	99.5
AST					
Aerobic exercise	−6.23 (−9.47, −3.00)	<0.05	6.3	0.0	52.2
Resistance exercise	−7.33 (−11.95, −2.72)	<0.05	26.9	0.1	67.9
HIIT	−5.97 (−12.95, 1.02)	>0.05	22.7	4.7	52.5
Mind-body exercise	−8.07 (−12.88, −3.25)	<0.05	44.1	0.1	76.1
GGT					
Aerobic exercise	−8.18 (-14.2, −2.13)	<0.05	5.2	0.2	50.5
Resistance exercise	−11.85 (-19.84,-3.87)	<0.05	22.8	0.0	71.1
HIIT	3.60 (-50.80, 58.00)	>0.05	24.2	56.4	32.4
Mind-body exercise	−13.77 (-22.00,-5.54)	<0.05	47.8	0.0	81.8

**FIGURE 5:**
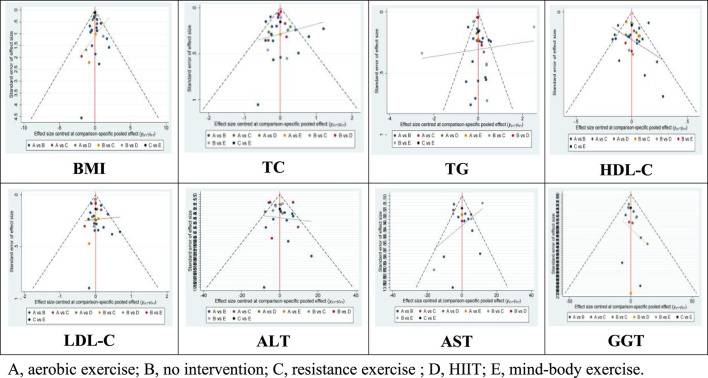
Funnel plots.

Moreover, the Wald test for inconsistency in the network did not yield significant results, indicating the absence of inconsistency among the included studies. Additionally, funnel plots did not reveal any evident bias (Figure 5).

#### 3.3.2 The intervention effect of exercise lipid metabolism in NAFLD

Lipid metabolism markers, including TC, TG, HDL-C, and LDL-in patients with NAFLD are important for assessing treatment outcomes. There are 32 studies assessing the impact of various exercise modalities on TC levels in NAFLD patients, 31 studies on TG, 30 on HDL-C, and 30 on LDL-C. Compared to the control, aerobic exercise, HIIT, resistance, and mind-body exercises significantly improve TC, TG, HDL-C, and LDL-C levels. Notably, mind-body exercises demonstrate the most substantial effect on TC (−0.67; 95% CI: −1.10 to −0.24; *p* < 0.05) and TG (−0.67; 95% CI: −1.10 to −0.24; *p* < 0.05), achieving the highest SUCRA scores of 89.7 for TC and 99.6 for TG. HIIT is most effective in enhancing HDL-C levels (0.12; 95% CI: 0.04 to 0.20; *p* < 0.05), with a SUCRA score of 76.1. Resistance training shows significant improvements in LDL-C (−0.20; 95% CI: −0.33 to −0.06; *p* < 0.05), with a SUCRA score of 69.3 (Table 2 and Figure 4). The Wald test for network inconsistency yielded no significant results, and funnel plots exhibited no discernible biases (Figure 5).

#### 3.3.3 Effects of exercise modalities on liver function in NAFLD

AST, ALT, and GGT are important biochemical markers for liver function tests and also serve as physiological bases for diagnosing NAFLD. Our review encompassed 20 studies on AST, 28 on ALT, and 15 on GGT. Among the different exercise modalities, mind-body exercises were found to significantly reduce AST (−8.07; 95% CI: −12.88 to −3.25; *p* < 0.05), ALT (−12.56; 95% CI: −17.54 to −7.58; *p* < 0.05), and GGT levels (−13.77; 95% CI: −22.00 to −5.54; *p* < 0.05). These interventions scored SUCRA values of 76.1 for AST, 99.5 for ALT, and 81.8 for GGT (Table 2 and Figure 4). Similar to the lipid metabolism studies, the Wald tests for inconsistency across the network of liver enzyme studies were not significant, and the funnel plots exhibited no discernible biases (Figure 5).

## 4 Discussion

This network meta-analysis assessed the efficacy of four distinct exercise modalities—aerobic, resistance, mind-body, and HIIT—on BMI in patients with NAFLD, alongside four lipid metabolism indicators (TC, TG, HDL-C, and LDL-C) and three liver function parameters (AST, ALT, and GGT). The comprehensive analysis substantiated the beneficial outcomes of these exercise forms in managing NAFLD, highlighting aerobic exercise as the most effective for BMI reduction. Resistance training showed superior results in LDL-C improvement, HIIT was most beneficial for HDL-C enhancement, and mind-body exercises showed significant efficacy in improving TC, TG, and liver function (AST, ALT, GGT). Existing literature has a varied evidence base, thereby advocating a cautious treatment of differing study designs and results in meta-analyses.

BMI, a crucial indicator associated with obesity and NAFLD (Camhi et al., 2011), was most effectively reduced through aerobic exercise in this analysis. The prolonged duration and moderate intensity of aerobic exercise might explain its efficacy in BMI reduction among NAFLD patients (Gao et al., 2021). Although high-intensity regimens have also been shown to decrease BMI (Zhu et al., 2020), aerobic exercise remains the focus of most research concerning NAFLD interventions. Moreover, aerobic exercise has demonstrated superior effects in ameliorating BMI and other NAFLD-related metrics such as glycated hemoglobin, resting blood pressure, and serum cholesterol levels (Kelley and Kelley, 2008). It also increases serum high-molecular-weight adiponectin, which influences insulin resistance and subsequently NAFLD (Haus et al., 2013). Moreover, compared to resistance exercise, aerobic exercise has a greater effect on reducing intrahepatic lipid content in patients with NAFLD (Orci et al., 2016; Slentz et al., 2011). Therefore, this study suggests that patients with NAFLD use aerobic exercise as a means of reducing BMI.

Our research indicates that resistance exercise is particularly effective in improving LDL-C levels. The likely mechanism underlying this benefit involves the enhancement of LDL receptor activity on hepatocyte membranes. This improvement in receptor activity increases the blood’s capacity to transport LDL-C, thereby lowering its concentration in the bloodstream (Chen et al., 2005). Furthermore, resistance exercise may facilitate the secretion of cytokines, known as myokines, from skeletal muscles. These myokines interact with various organs to mediate metabolic processes (Benatti and Pedersen, 2015; Karstoft and Pedersen, 2016). Notably, the secretion of myokine Irisin during resistance exercise is associated with the induction of browning in subcutaneous fat cells, which enhances thermogenesis and energy expenditure (Boström et al., 2012). Kim et al. (2016) observed a significant increase in circulating Irisin levels during resistance exercises, with negligible changes in aerobic exercises, highlighting Irisin’s potential role in improving NAFLD outcomes. Additionally, exercise may enhance insulin sensitivity and upregulate LDL-C mRNA expression, thereby accelerating LDL-C metabolism (Young and Stout, 1987). In line with previous findings, regular exercise has been shown to improve LDL-C markers in patients with NAFLD (Fu et al., 2018).

HIIT, which alternates between high-intensity exercise and lower-intensity recovery periods. Our findings indicate that HIIT is most effective in improving HDL-C levels. Previous studies have identified exercise intensity as a significant factor in the variations observed in HDL-C levels (Gao et al., 2021). Notably, studies reporting significant differences in HDL-C levels between control and experimental groups also observed higher exercise intensities (Wang et al., 2020). Despite these benefits, we recommend that individuals select an exercise regimen that is manageable over the long term. HIIT, while beneficial, may not be suitable for all populations, particularly the elderly, due to its demanding nature. This study has yielded a novel finding: mind-body exercises demonstrate superior efficacy in improving liver function markers (AST, ALT, GGT) and certain lipid metabolism indicators (TC, TG) compared to three other forms of exercise.

Most of the Mind-body exercises belong to low-intensity aerobic exercises such as yoga, tai chi, and baduanjin. In addition to its ability to increase energy expenditure and promote fat metabolism, a unique aspect of mind-body exercise is that it focuses on the unity of mind and body integrating physical movement with psychological processes. Exercise has been shown to reduce levels of TC and TG (Yu et al., 2017; Costa et al., 2020), yet the underlying mechanisms remain unclear. Some studies suggest that exercise increases the activity of lipolytic enzymes and accelerates the rate of breakdown into mitochondrial energy supply (Bianchi et al., 2021), while others associate it with adipocyte factors such as leptin and adiponectin (Izadi et al., 2013). However, the means by which exercise improves adipose factors are not yet clear, necessitating further research for confirmation. AST, ALT, and GGT are critical markers for identifying liver function and are closely related to NAFLD. Studies reporting improvements in ALT, AST, and GGT through exercise align with our findings (Mascaró et al., 2022; Gao et al., 2021).

In addition to exercise interventions, other methods such as dietary management and pharmacological treatments are effective strategies for managing NAFLD. Controlling intake is a crucial aspect of dietary intervention. When the body consumes excessive amounts of saturated and unsaturated fatty acids, there is a significant impact on hepatic fat accumulation (Rosqvist et al., 2014). However, there remains ongoing debate regarding optimal dietary composition and patterns for NAFLD management (El-agroudy et al., 2019; Moore et al., 2020). Pharmacological treatments predominantly target disease-related pathways, including pathogenic factors and associated metabolic disorders (Rong et al., 2023). Due to the complex pathogenesis of NAFLD, specific pharmacological treatments are still lacking (Rong et al., 2023; Romero-Gómez et al., 2017). Compared to these interventions, exercise appears more favorable, as most RCTs have demonstrated that exercise confers significant benefits for NAFLD (Nassir, 2022). Studies have shown that exercise reduces hepatic steatosis, improves metabolic function, and ameliorates fibrosis (Huber et al., 2019; O’Gorman et al., 2020). Various studies have explored the underlying mechanisms of exercise in NAFLD management. Some research suggests that exercise regulates hepatic fatty acid synthesis and oxidation, as well as mitochondrial structure, thereby preventing liver damage (Oh et al., 2021; Rector et al., 2011; Thyfault and Rector, 2020). Additionally, exercise has been found to improve hepatic inflammation, partly by increasing sirtuin (SIRT) activity (Bianchi et al., 2021). Furthermore, exercise enhances peripheral insulin sensitivity, which in turn reduces hepatic lipogenesis (Cuthbertson et al., 2016). Beyond these physiological mechanisms, the psychological benefits of exercise have also been explored. Physical and mental exercises, such as tai chi and Yoga, can reduce anxiety, depression, and stress (Dong et al., 2024). Consequently, some studies suggest that such exercises may improve NAFLD progression by alleviating stress and anxiety, improving insulin sensitivity and hepatic fat accumulation (Shomaker et al., 2010), and regulating hormone levels, including reducing cortisol (Targher et al., 2006). However, in clinical practice, NAFLD patients often demonstrate low readiness for behavioral change and may lack motivation to adopt healthier lifestyles (Centis et al., 2013). Moreover, long and demanding exercise regimens may pose adherence challenges for these patients.

In summary, the reason mind-body exercises excel in improving liver function and lipid profile markers in NAFLD may be due to their comprehensive action on both the physical and psychological levels, promoting health through various mechanisms including but not limited to stress management, psychological health improvement, endocrine and metabolic function regulation, as well as enhanced antioxidative and anti-inflammatory effects. This suggests that a holistic treatment approach may be more effective than singular exercise modalities in managing metabolic diseases like NAFLD. Therefore, we recommend incorporating mind-body exercise as a viable exercise modality in the treatment of NAFLD, and can also be combined with diet as an adjunct to the treatment of NAFLD.

## 5 Limitations and future direction

There are several noteworthy limitations in this study. Firstly, the study compared different types of exercise without strict regulations regarding exercise volume, intensity, duration, and age. The inconsistency in the methods of measuring exercise intensity across the studies and the lack of standardization may introduce a certain degree of bias to the results. Furthermore, the study encompassed research that investigated the combined effects of medication and exercise interventions. This aspect raises the possibility of synergistic effects influencing the outcomes, thereby introducing an additional layer of complexity and potential bias in interpreting the results. The final limitation arises from the specific geographic region; for instance, the mind-body exercises included in this study all originate from Asia. This is mainly due to the fact that mind-body exercises originated in Asia. However, this inadvertently increases the potential influence of cultural, genetic, and environmental factors on exercise interventions. It is important to note that the prevalence of NAFLD also varies significantly across different geographic regions. In light of these considerations, it is essential to approach the study’s conclusions with a degree of caution. This is because they may pose challenges to the overall validity and reliability of the study’s results.

To mitigate biases, future investigations should establish more stringent requirements for various exercise types. Further research is also needed to determine whether these findings can be replicated in different racial populations to understand potential race-specific responses to different exercise modalities. Additionally, combining different exercise modalities or integrating them with pharmacological interventions could provide deeper insights into their effects on NAFLD patients. Finally, there is a need for more in-depth research and exploration of the potential mechanisms underlying the therapeutic effects of different exercise modalities on NAFLD.

## 6 Conclusion

Our network meta-analysis revealed that aerobic exercise significantly enhances BMI in individuals with NAFLD, resistance exercise was found to be particularly effective in improving LDL-C, whereas HIIT markedly boosts HDL-C. Additionally, mind-body exercises were superior in enhancing TC, TG, and key liver function parameters (AST, ALT, GGT). Based on these findings, it is recommended that NAFLD patients incorporate mind-body exercises into their treatment regimen to optimize health outcomes.

## Data Availability

The original contributions presented in the study are included in the article/Supplementary Material, further inquiries can be directed to the corresponding author.
