# Supplementary material for: Comparative efficacy of different exercise modalities on metabolic profiles and liver functions in non-alcoholic fatty liver disease: a network meta-analysis
Source: Front Physiol. 2024 Sep 11;15:1428723. doi: 10.3389/fphys.2024.1428723 (PMC11457013; doi:10.3389/fphys.2024.1428723)
Supplement: Supplementary file 1 [file Table1.docx]

Supplementary Material

**Comparative efficacy of different exercise modalities on metabolic profiles and liver functions in non-alcoholic fatty liver disease: a network meta-analysis**

**S1. Search strategies**

**S2. Definition of intervention modalities**

**S3. Fig.1 Risk of bias in individual studies. +, low risk of bias; ?, unclear risk of bias; –, high risk of bias**

**S4.** **Registration and protocol**

**S1. Search strategies**

(“NAFLD”, “exercise OR NAFLD”, “training OR NAFLD”, “sport OR NAFLD”, “physical activity OR NAFLD” ,“exercise OR physical OR NAFLD” ,“exercise OR training OR NAFLD”, “aerobic OR NAFLD”, “aerobic OR sport OR NAFLD” , “aerobic exercise OR NAFLD” , “aerobic training OR NAFLD”, “resistance OR NAFLD”, “resistance training OR NAFLD”, “resistance exercise OR NAFLD”, “mind-body, NAFLD”, “mind-body OR sport OR NAFLD” , “mind-body exercise OR NAFLD”, “mind-body OR training OR NAFLD”, “mind-body OR exercise OR NAFLD”, “high-intensity interval training OR NAFLD”, “HIIT OR NAFLD”, “high-intensity interval training OR exercise OR NAFLD”, “strength training OR NAFLD”, “strength training OR exercise OR NAFLD”, “tai chi OR NAFLD”, “baduanjin OR exercise, NAFLD” OR “NAFLD, wuqinxi exercise”, “yoga OR NAFLD”, “exercise OR obesity OR NAFLD”, “training OR obesity OR NAFLD”, “sport OR obesity OR NAFLD”, “physical activity OR obesity OR NAFLD”, “lifestyle OR NAFLD”, “exercise OR lifestyle OR NAFLD”, “training OR lifestyle OR NAFLD”, “sport OR lifestyle OR NAFLD”, “physical activity OR lifestyle OR NAFLD”, “exercise OR liver OR NAFLD”, “training OR liver OR NAFLD”, “sport OR liver OR NAFLD”, “physical activity OR liver OR NAFLD”)

**S2. Definition of intervention modalities**

**Aerobic exercise**: duration of 15 minutes or more with heart rate maintained between 60% and 80% of maximum heart rate.

Includes: walking or jogging; cycling; cardio equipment (a rowing machine; stair climbers; an elliptical; a treadmill); swimming and other forms of exercise

**Resistance exercise**: an active movement performed by a muscle to overcome external resistance.

Includes: weight lifting, push-ups, dumbbells, barbells, pull-ups, resistance bands and other training modalities

**High-intensity interval training (HIIT)**, which divides exercise into high-intensity intervals and recovery periods.

The article clearly mentions using HIIT.

**Mind-body exercise**: mind-body exercise involves a variety of actions such as stretching and relaxation of skeletal muscles, as well as coordinated body and regular breathing movements. Additionally, meditative states are also involved in mind-body exercises to regulate attention and consciousness, focusing on the unity of mind and body, it is an activity that combines physical movement with mental processes.

Includes: tai chi, qigong, yoga, pilates, baduanjin, and wuqinxi.

**S3. Fig.1 Risk of bias in individual studies. +, low risk of bias; ?, unclear risk of bias; –, high risk of bias**


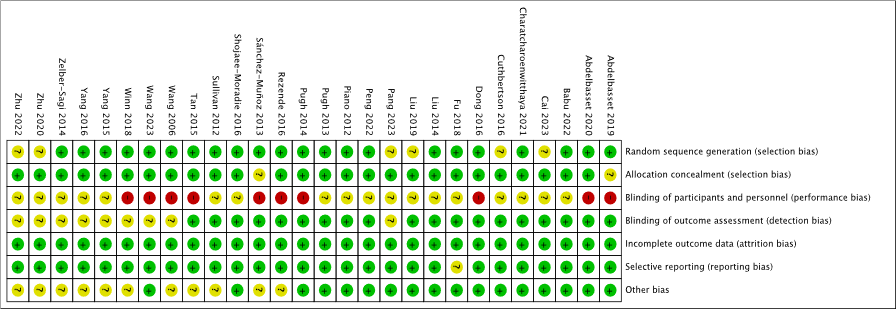


**S4.** **Registration and protocol**

Q1, The review protocol can be accessed (https://www.crd.york.ac.uk/prospero/#recordDetails)

Q2, No corrections have been made to the information provided in the registration or review protocol.
