# Supplementary material for: Comparative efficacy of different exercise modalities on metabolic profiles and liver functions in non-alcoholic fatty liver disease: a network meta-analysis
Source: Front Physiol. 2024 Sep 11;15:1428723. doi: 10.3389/fphys.2024.1428723 (PMC11457013; doi:10.3389/fphys.2024.1428723)
Supplement: Supplementary file 2 [file Table2.docx]

| **Section and Topic** | **Item #** | **Checklist item** | **Location where item is reported** |
| --- | --- | --- | --- |
| **TITLE** | | |  |
| Title | 1 | The report is Identified as a meta-analysis and network meta-analysis systematic review. | Title |
| **ABSTRACT** | | |  |
| Abstract | 2 | The summary contains all information in the PRISMA 2020 for Abstracts checklist, and the registration code is CRD42024526332. | Abstract |
| **INTRODUCTION** | | |  |
| Rationale | 3 | Described in the introduction. | Introduction |
| Objectives | 4 | Described in the introduction. | Introduction |
| **METHODS** | | |  |
| Eligibility criteria | 5 | Described in the methods. (Specify the inclusion and exclusion criteria for the review based on the PICOS principles.) | Methods |
| Information sources | 6 | Described in the methods. (Specify all databases, registers, websites, organizations, reference lists, and other sources searched or consulted to identify studies. Specify the date when each source was last searched or consulted.) | Methods |
| Search strategy | 7 | Described in the methods. (Present the full search strategies for all databases, registers, and websites, including any filters and limits used.) | Methods |
| Selection process | 8 | Described in the methods. (The methods used to decide whether a study met the inclusion criteria of the review, including how many reviewers screened each record and each report retrieved, whether they worked independently, and if applicable, details of automation tools used in the process.) | Methods |
| Data collection process | 9 | Described in the methods. (Specify the methods used to collect data from reports, including how many reviewers collected data from each report, whether they worked independently, any processes for obtaining or confirming data from study investigators, and if applicable, details of automation tools used in the process) | Methods |
| Data items | 10a | Described in the methods. (List and define all outcomes for which data were sought. Specify whether all results that were compatible with each outcome domain in each study were sought (e.g. for all measures, time points, analyses), and if not, the methods used to decide which results to collect.) | Methods |
|  | 10b | Described in the methods. (List and define all other variables for which data were sought (e.g. participant and intervention characteristics, funding sources). Describe any assumptions made about any missing or unclear information.) | Methods |
| Study risk of bias assessment | 11 | Described in the methods. (Specify the methods used to assess the risk of bias in the included studies, including details of the tool(s) used, how many reviewers assessed each study and whether they worked independently, and if applicable, details of automation tools used in the process.) | Methods |
| Effect measures | 12 | Described in the methods. (Specify for each outcome the effect measure(s) (e.g. risk ratio, mean difference) used in the synthesis or presentation of results.) | Methods |
| Synthesis methods | 13a | Described in the methods. (Describe the processes used to decide which studies were eligible for each synthesis (e.g. tabulating the study intervention characteristics and comparing against the planned groups for each synthesis (item #5)).) | Methods |
|  | 13b | Described in the methods. (Describe any methods required to prepare the data for presentation or synthesis, such as handling of missing summary statistics, or data conversions.) | Methods |
|  | 13c | Described in the methods. (Describe any methods used to tabulate or visually display results of individual studies and syntheses.) | Methods |
|  | 13d | Described in the methods. (Describe any methods used to synthesize results and provide a rationale for the choice(s). If meta-analysis was performed, describe the model(s), method(s) to identify the presence and extent of statistical heterogeneity, and software package(s) used.) | Methods |
|  | 13e | Described in the methods. (Describe any methods used to explore possible causes of heterogeneity among study results (e.g. subgroup analysis, meta-regression).) | Methods |
|  | 13f | Described in the methods. (Describe any sensitivity analyses conducted to assess the robustness of the synthesized results） | Methods |
| Reporting bias assessment | 14 | Described in the methods. (Describe any methods used to assess risk of bias due to missing results in a synthesis (arising from reporting biases).) | Methods |
| Certainty assessment | 15 | Described in the methods. (Describe any methods used to assess certainty (or confidence) in the body of evidence for an outcome.) | Methods |
| **RESULTS** | | |  |
| Study selection | 16a | Described in the results. (The results of the search and selection process, from the number of records identified in the search to the number of studies included in the review, using a flow diagram.) | Results |
|  | 16b | Described in the results. (Cite studies that might appear to meet the inclusion criteria, but which were excluded, and explain why they were excluded.) | Results |
| Study characteristics | 17 | Described in the results. (Included study and present its characteristics.) | Results |
| Risk of bias in studies | 18 | Described in the results. (Present assessments of risk of bias for each included study.) | Results |
| Results of individual studies | 19 | Described in the results. (For all outcomes, present, for each study: (a) summary statistics for each group (where appropriate) and (b) an effect estimates and its precision (e.g. confidence/credible interval), ideally using structured tables or plots.) | Results |
| Results of syntheses | 20a | Described in the results. (For each synthesis, briefly summarize the characteristics and risk of bias among contributing studies.) | Results |
|  | 20b | Described in the results. (The results of all statistical syntheses conducted. If meta-analysis was done, present for each the summary estimate and its precision (e.g. confidence/credible interval) and measures of statistical heterogeneity. If comparing groups, describe the direction of the effect.) | Results |
|  | 20c | Described in the results. (The results of all investigations of possible causes of heterogeneity among study results.) | Results |
|  | 20d | Described in the results. (Present results of all sensitivity analyses conducted to assess the robustness of the synthesized results.） | Results |
| Reporting biases | 21 | Described in the results. (The assessments of risk of bias due to missing results (arising from reporting biases) for each synthesis assessed.) | Results |
| Certainty of evidence | 22 | Described in the results. (The assessments of certainty (or confidence) in the body of evidence for each outcome assessed.) | Results |
| **DISCUSSION** | | |  |
| Discussion | 23a | Described in the discussion. (A general interpretation of the results in the context of other evidence.) | Discussion |
|  | 23b | Described in the discussion. (Discuss any limitations of the evidence included in the review.) | Discussion |
|  | 23c | Described in the discussion. (Discuss any limitations of the review processes used.) | Discussion |
|  | 23d | Described in the discussion. (Discuss implications of the results for practice, policy, and future research.) | Discussion |
| **OTHER INFORMATION** | | |  |
| Registration and protocol | 24a | Registration number CRD42024526332. | Methods |
|  | 24b | Described in the Support information. The review protocol can be accessed.( https://www.crd.york.ac.uk/prospero/#recordDetails) | Support information |
|  | 24c | Described in the Support information. No corrections have been made to the information provided in the registration or review protocol | Support information |
| Support | 25 | Explained in the funding. (No funding) | Funding |
| Competing interests | 26 | Explained in the author contributions. (No conflict of interest) | Author contributions |
| Availability of data, code and other materials | 27 | Described in the Support information. Upload as a file to the support file. (Report which of the following are publicly available and where they can be found: template data collection forms; data extracted from included studies; data used for all analyses; analytic code; any other materials used in the review.) | Support information |

For more information, visit: <http://www.prisma-statement.org/>
